# Ultra-broadband and wide-angle nonreciprocal thermal emitter based on Weyl semimetal metamaterials

**DOI:** 10.1515/nanoph-2023-0520

**Published:** 2024-01-09

**Authors:** Kezhang Shi, Yuwei Sun, Run Hu, Sailing He

**Affiliations:** Ningbo Innovation Center, Zhejiang University, Ningbo 315100, China; Taizhou Hospital, Zhejiang University, Taizhou, China; Centre for Optical and Electromagnetic Research, National Engineering Research Center for Optical Instruments, Zhejiang University, Hangzhou 310058, China; School of Energy and Power Engineering, Huazhong University of Science and Technology, Wuhan 430074, China; Department of Electrical Engineering, Royal Institute of Technology, SE-100 44 Stockholm, Sweden

**Keywords:** nonreciprocal thermal radiation, mid-infrared broadband, wide-angle range, Weyl semimetals

## Abstract

Nonreciprocal thermal radiation can violate Kirchhoff’s law and exhibit different emissivity at symmetric polar angles relative to the normal direction. Realizing a mid-infrared broadband nonreciprocal thermal emitter with a wide emission angle range is a fundamental yet challenging task, particularly without the need for an external magnetic field. Here, we propose a nonreciprocal thermal emitter operating in the mid-infrared that achieves a significantly nonreciprocal thermal radiation in a wavelength range from 12 μm to 20 μm, spanning a wide angular range from 16° to 88°. This is achieved by utilizing a multilayered Weyl semimetal (WSM)/dielectric structure, which takes the advantage of the strong nonreciprocity of WSMs with different Fermi levels and epsilon-near-zero-induced Brewster modes. The results provide a wider angular range in the broad mid-infrared band compared to previous attempts. The robustness of the nonreciprocal radiation is confirmed through wavelength-averaged emissivity across the azimuth angle *φ* range from 0° to 360°. Some possible materials and nanostructures as dielectric layers are discussed, showcasing the flexibility and reliability of the design. This work holds promising potential applications such as enhanced radiative cooling, thermal emitters for medical sensing and infrared heating, energy conversion, etc.

## 1 Introduction

Metamaterials, such as multilayered structures composed of alternative lossy metal-dielectric slabs [1], [2], resonators with multiband response [3], [4], [5], or complex epsilon-near-zero (ENZ) films [6], [7], [8], [9], have been explored for achieving mid-infrared (mid-IR) broadband emission and absorption with omnidirectional or some specific angle range. The ability to control broadband thermal emission in the mid-IR range is important for critical applications including radiative cooling [10]–[14], energy conversion [15]–[19], medical sensing and infrared heating [20], infrared (IR) camouflage [21], [22], and thermal sources for multiple gas/optical sensing [23]. However, previous studies based on reciprocal materials have encountered a fundamental limitation where equal emissivity/absorptivity is observed at two symmetric polar angles relative to the normal direction, which poses an obstacle to achieving higher energy conversion efficiency [24]. It is a fundamental interest to overcome this challenge and realizing a nonreciprocal thermal emitter in both broadband and a wide emission angle range, which holds promising potential applications in higher-efficiency radiative cooling, thermophotovoltaic systems, directional thermal camouflage, etc.

Kirchhoff’s law states that the directional spectral emissivity of reciprocal materials is equal to their directional spectral absorptivity for both transverse electric (TE) and transverse magnetic (TM) polarizations. However, recent works [25], [26], [27] have demonstrated that nonreciprocal materials, characterized by permittivity tensors with opposite non-zero off-diagonal components, can violate Kirchhoff’s law. This means that the directional spectral emissivity can differ from the directional spectral absorptivity. Magneto-optical (MO) materials, such as InAs and InSb, exhibit nonreciprocal thermal radiation/absorption in the mid-IR when subjected to an external magnetic field. The nanostructures of these materials can support magneto-optical lattice resonance [28] or guided mode resonances [26], [29], enabling directional nonreciprocal thermal radiation at specific angles (e.g., ±1° or other larger angles). However, these effects are typically limited to narrowband operating wavelengths and require significant magnetic fields. To achieve broadband nonreciprocal thermal radiation, one can utilize multilayered epsilon-near-zero (ENZ) films that support Berreman mode [27], [30]. Although ENZ materials (such as polar materials with phonons absorption) are known to support such leaky *p*-polarized electromagnetic modes near the material resonance pole, their functionality is usually limited to a certain range of incident angles (typically at large oblique angles, such as >70° for some thin film). Note that this resonance mode, existing within the light line, is also recognized as the Brewster mode in the context of metallic materials characterized by absorption originating from free carriers [26], [31].

Weyl semimetals (WSMs) have gained significant attention due to their ability to exhibit highly unusual and remarkably large gyrotropic optical responses in the mid-IR. These responses, characterized by off-diagonal components comparable in magnitude to the diagonal components, stem from the unique topologically nontrivial electronic states and inherent time-reversal symmetry breaking of WSMs [32], [33], [34]. WSMs represent a recently discovered class of three-dimensional gapless topological materials that possess accidental degenerate points in their band structure known as Weyl nodes. These nodes host chiral Fermions and appear in pairs with opposite chirality [32]. Each Weyl node serves as a source or drain of Berry curvature in the momentum space. The momentum separation (denoted as 2**b**) between Weyl nodes acts similar to an internal magnetic field, enabling the prediction of nonreciprocal thermal radiation without the need for external magnetic field [30], [32], [35], [36]. Recent studies have demonstrated pronounced nonreciprocal thermal radiation within the wavelength range of 14.5–16.5 μm, occurring at angles between 36° and 64.5°, using a cascaded dielectric and WSMs grating structure [37]. Another investigation has revealed that a concise trilayer configuration comprising WSM/tungsten (W)/germanium (Ge) enabled nonreciprocal thermal radiation over a broad wavelength range and at multiple angles [38]. Despite the presence of dielectric layers aids in broadening the bandwidth, some wavelengths and angles still lack continuous and conspicuous nonreciprocity when employing a single WSM. Therefore, achieving nonreciprocal emission with both ultra-broadband characteristics and a wide-angle range (e.g., spanning from 0° to 90°) without relying on an external magnetic field remains a critical and challenging task.

Herein, we present a mid-IR ultra-broadband nonreciprocal thermal emitter with a wide emission angle, achieved by utilizing magnetic WSMs/dielectric multilayer films. (i) The directional spectral emissivity is investigated across a broad wavelength band from 12 μm to 20 μm, covering a wide angular range from −88° to 88°. The ultra-broadband and asymmetric angular emissivity is explained by the ENZ-induced Brewster modes and the intrinsic nonreciprocity resulting from the two WSMs with different Fermi levels. The wide angular nonreciprocity is also attributed to the presence of a lossless dielectric layer, which acts as an additional cavity, thereby enhancing emission/absorption at smaller angles. (ii) The wavelength-averaged emissivity is studied with arbitrary azimuth angle ranges from 0° to 360°. The significant difference between the emissivity and absorptivity in each plane indicates the robustness of the nonreciprocal thermal radiation. (iii) The alternative materials with nanostructures as the dielectric layers are discussed, demonstrating the flexibility of our design scheme and the reliable performance of the nonreciprocal emitter. This work has practical implications for the development of wide emission angle broadband nonreciprocal devices, which can find applications in various fields such as radiative cooling, energy conversion, and thermal management.

**Figure 1:**
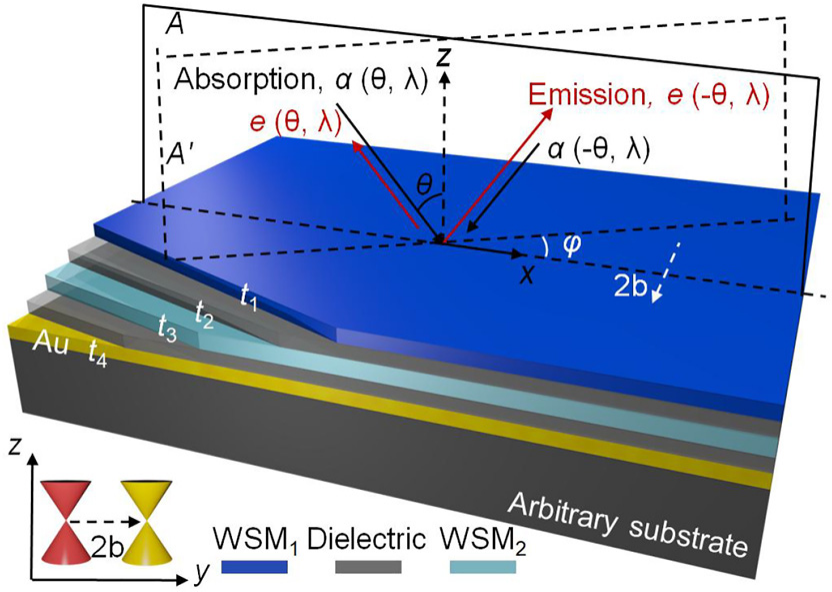
A schematic illustration of the WSM/dielectric metamaterials consisting of two WSM layers with different Fermi levels and alternating dielectric films on a gold reflector. *t*_1_ = *t*_2_ = 0.2 μm, *t*_3_ = 0.3 μm, and *t*_4_ = 0.1 μm are the corresponding thicknesses of the films. *φ* is the azimuth angle of plane *A*′, and *θ* is the polar angles in each incident planes. The inset shows the electronic band structures of the two WSM layers, with two Weyl nodes of opposite chirality separated by 2**b** in the momentum space. These nodes act as an internal magnetic field and are assumed to align along the opposite direction of the *y*-direction.

The multilayer structure shown in Figure 1 consists of alternating layers of WSM and dielectric film. The two WSM layers, labeled as WSM_1_ and WSM_2_, have different Fermi levels. The thicknesses of WSM_1_, dielectric film, WSM_2_, and Au are chosen as *t*_1_ = *t*_2_ = 0.2 μm, *t*_3_ = 0.3 μm, and *t*_4_ = 0.1 μm, respectively. The substrate can be any material as the electromagnetic field is blocked by the Au reflector. The incident plane in the *x*–*z* plane is denoted as *A*, and the incident plane at an azimuth angle *φ* is denoted as *A*′. When *φ* = 0°, plane *A*′ coincides with plane *A*. The momentum separation (denoted as 2**b**) of the Weyl nodes in the WSM can be considered as an axial vector that behaves similarly to an internal magnetic field. By choosing the direction along the opposite direction of the *y*-axis, the permittivity tensor of the WSM can be expressed as [39]:

(1) $$\bar{\bar{\varepsilon }}=\left[\begin{array}{ccc} \varepsilon_{w} & 0 & -i\varepsilon_{a}\\ 0 & \varepsilon_{w} & 0\\ i\varepsilon_{a} & 0 & \varepsilon_{w} \end{array}\right]$$

where the components are:

(2) $$\varepsilon_{w}=\varepsilon_{b}+i\frac{\sigma }{\Omega_{0}}$$

(3) $$\varepsilon_{a}=\frac{-be^{2}}{2\pi^{2}\hbar \omega \varepsilon_{0}}$$

It should be noted that Equation (1) is deduced by employing a coordinate transformation to address the permittivity tensor *ε* as referenced in Ref. [39]. In this context, $\bar{\bar{\varepsilon }}$ = *M* ^*T*^ *εM*, wherein *M* represents a conventional right-handed coordinate system rotation matrix about the *x*-axis with a rotation angle *κ* = −90°, and *M* ^*T*^ denotes the transposed matrix.

(4) $$M=\left[\begin{array}{ccc} 1 & 0 & 0\\ 0 & \cos\left(\kappa \right) & \sin\left(\kappa \right)\\ 0 & -\sin\left(\kappa \right) & \cos\left(\kappa \right) \end{array}\right]$$

In Equations (2) and (3), *ε*_*b*_ represents the background permittivity, and Ω_0_ = *ℏω*/*E*_F_ is the normalized real frequency. We have examined the performance of the nonreciprocal thermal radiation (not shown) when 2**b** for WSM_1_ and WSM_2_ have opposite signs. For emissivity/absorptivity, altering the sign of 2**b** has the same effect as changing the emission/absorption angle’s sign, i.e., *e* (*θ*, −2**b**) = *e* (−*θ*, 2**b**), and *α* (*θ*, −2**b**) = *α* (−*θ*, 2**b**). This behavior bears similarity to the effects observed when manipulating the direction of an applied external magnetic field in magneto-optical materials, as detailed in Ref. [26]. Here, the opposite signs of 2b for the two WSM layers (e.g., −2**b** for WSM_1_ and +2**b** for WSM_2_) does not enable the generation of a continuous wide-angle (e.g., from 1° to 89° or −1° to −89°) with higher emissivity on the same side with respect to the normal direction, which hinders the realization of ultra-broadband and wide-angle non-reciprocal thermal radiation. Therefore, in this work, the sign of 2**b** in each WSM layer is identical. *σ* denotes the bulk conductivity, which is calculated using the random phase approximation to a two-band model with spin degeneracy. In this approximation model both interband and intraband transitions are considered and we have [40], [41].

(5) $$\begin{array}{ll} \sigma & =\frac{r_{s}g}{6}\Omega G\left(\frac{\Omega }{2}\right)+i\frac{r_{s}g}{6\pi }\left\{\frac{4}{\Omega }\left[1+{\frac{\pi }{3}}^{2}{\left(\frac{k_{B}T}{E_{F}}\right)}^{2}\right]\right.\\ & \;\left.+8\Omega \int_{0}^{\xi_{c}}\frac{G\left(\xi \right)-G\left(\frac{\Omega }{2}\right)}{\Omega^{2}-4\xi^{2}}\xi d\xi \right\} \end{array}$$

*r*_*s*_ = *e* ^2^/4π*ε*_0_ *ℏV*_F_ is the effective fine structure constant, *ε*_0_ = 8.8542 × 10^−12^ F m^−1^ is the permittivity of vacuum, *V*_F_ is the Fermi velocity, *g* is the number of Weyl points. Ω = *ℏ*(*ω* + *iτ* ^−1^)/*E*_F_ is the normalized complex frequency, and *G*(*E*) = *n*(−*E*) – *n*(*E*), where *n*(*E*) = 1/[exp((*E* − *E*_F_)/*k*_B_ *T*) + 1] is the Fermi distribution function. Similar to that of graphene [17], [40], [42]–[44], *G*(*E*) can be expressed as:

(6) $$\begin{array}{ll} G\left(E\right) & =n\left(-E\right)-n\left(E\right)\\ & =\sin h\left(\frac{E}{k_{B}T}\right)/\left[\cos h\left(\frac{E_{F}}{k_{B}T}\right)+\cos h\left(\frac{E}{k_{B}T}\right)\right] \end{array}$$

In equations (5) and (6), *ξ* = *E*/*E*_F_ is a normalized energy, *ξ*_c_ = *E*_c_/*E*_F_, where *E*_c_ is the cutoff energy beyond which the band dispersion is no longer linear [40], and *E*_F_ is the Fermi level. According to ref. [30], we choose *ε*_b_ = 6.2, *V*_F_ = 0.83 × 10^5^ m/s, *g* = 2, *τ* = 450 fs, *ξ*_c_ = 3, and *b* = 8.5 × 10^8^ m^−1^ in the calculation. When there is a non-zero momentum separation, the permittivity tensor of the WSM exhibits non-zero off-diagonal components that are opposite in sign. This leads to the breaking of Lorentz reciprocity. In this study, the Fermi levels of WSM_1_ and WSM_2_ are set to 61 meV and 88 meV, respectively. The dielectric layers are modeled as planar structures with a vacuum filling ratio denoted as *f*_vac_, and an effective medium method is employed [45], [46]:

(7) $${\bar{\bar{\varepsilon }}}_{\text{die}}=\left[\begin{array}{ccc} \varepsilon_{⊥} & 0 & 0\\ 0 & \varepsilon_{⊥} & 0\\ 0 & 0 & \varepsilon_{\|} \end{array}\right]$$

where the components are:

(8) $$\varepsilon_{⊥}=\frac{\varepsilon_{\text{host}}\left[\varepsilon_{\text{host}}\left(1-f_{\text{vac}}\right)+1+f_{\text{vac}}\right]}{\varepsilon_{\text{host}}\left(1+f_{\text{vac}}\right)+1-f_{\text{vac}}}$$

(9) $$\varepsilon_{\|}=f_{\text{vac}}+\left(1-f_{\text{vac}}\right)\varepsilon_{\text{host}}$$

*f*_vac_ takes a value of 0 or 1, indicating whether the dielectric layer consists of the pure host material or vacuum, respectively. The permittivity of Au is described by the Drude model with a plasma frequency of 1.32 × 10^16^ rad s^−1^ and relaxation rate of 1.2 × 10^14^ rad s^−1^ [47].

For planar structures with an optically thick substrate (e.g., the Au reflector), the transmission *T* is negligible. As a result, in each plane rotated around the *z*-axis, the directional spectral absorptivity *α* (*φ*, *θ*, *λ*) and emissivity *e* (*φ*, *θ*, *λ*) can be related to the reflectivity *R* (*φ*, *θ*, *λ*) through the following simple relationships [28]

(10) α(φ,θ,λ)=1−R(φ,θ,λ)

(11) e(φ,θ,λ)=1−R(φ,−θ,λ)

Based on equations (10) and (11), the emissivity and absorptivity can be simply related by:

(12) e(φ,θ,λ)=α(φ,−θ,λ)

In this work, *R* (*φ*, *θ*, *λ*), *e* (*φ*, *θ*, *λ*), *α* (*φ*, *θ*, *λ*) of both the TM and TE polarizations are calculated by rigorous coupled wave analysis (RCWA) [48] for arbitrary plane with different azimuth angle *φ*. For TM polarization, we consider *R*_*p*_ (*φ*, *θ*, *λ*) = *R*_*pp*_ (*φ*, *θ*, *λ*) + *R*_*ps*_ (*φ*, *θ*, *λ*), and for TE polarization, we consider *R*_*s*_ (*φ*, *θ*, *λ*) = *R*_*ss*_ (*φ*, *θ*, *λ*) + *R*_*sp*_ (*φ*, *θ*, *λ*), where *R*_*ps*_ and *R*_*sp*_ are the corresponding polarization conversions of the reflectivity. To validate the results, comparisons between the codes and the finite element method (FEM) simulations using COMSOL Multiphysics have been conducted.

## 2 Results and discussions

**Figure 2:**
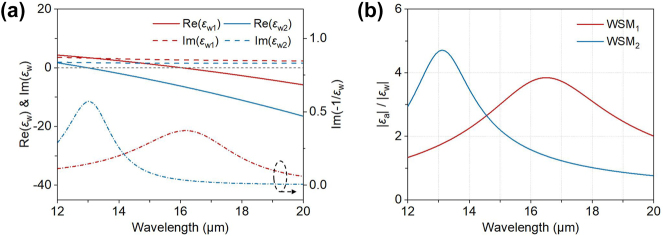
Analysis of the permittivity tensor of the two WSMs. (a) The solid and dashed lines represent the real and imaginary parts of *ε*_w_ of WSM materials, respectively. The dash-dotted lines indicate the wavelengths of the Brewster mode obtained from the energy-loss function Im(−1/*ε*_w_). (b) The ratio of permittivity components |*ε*_a_| to |*ε*_w_| within the wavelength range of 12–20 μm for the two WSM layers. The two peaks around 13.01 μm and 16.15 μm correspond to the largest nonreciprocity in this wavelength range, attributed to the ENZ properties.

Figure 2a shows the real and imaginary parts of *ε*_w_, showcasing that the two WSM layers have near-zero permittivity at specific wavelengths, which decreases with increasing Fermi level. The ENZ film, regarded as a metallic material due to its *ε*_w_ being accurately characterized by a conventional lossy Drude model [39], when interfaced with metal, induces a leaky *p*-polarized electromagnetic mode, known as Brewster mode [31]. The frequency of the resonance mode will decrease and approach the plasma frequency when the thickness of the ENZ film decreases, with the same behaviors as the Berreman mode (which is observed near the resonance of the longitudinal optical phonon polariton in materials exhibiting phonon absorption). This mode can couple to free space light at specific oblique angles. Its wavelength can be estimated by the peaks (dashed-dotted lines in Figure 2a) of the energy-loss function Im(−1/*ε*_w_) [7]. In our case, the wavelengths are 13.01 μm and 16.15 μm for WSM_1_ and WSM_2_, respectively. Consequently, stacking ENZ films allows for broadband directional thermal radiation within the wavelength range of 12 μm–20 μm. Figure 2b illustrates the ratio of |*ε*_a_| to |*ε*_w_| within this wavelength range. As expected, two peaks are observed near the Brewster wavelengths, and the relatively large ratio signifies the strong nonreciprocity. By harnessing the advantageous features of both the ENZ and nonreciprocal properties of the two WSM layers, we propose a multilayer structure depicted in Figure 1, aiming to realize a broadband and wide-angle nonreciprocal thermal emitter.

**Figure 3:**
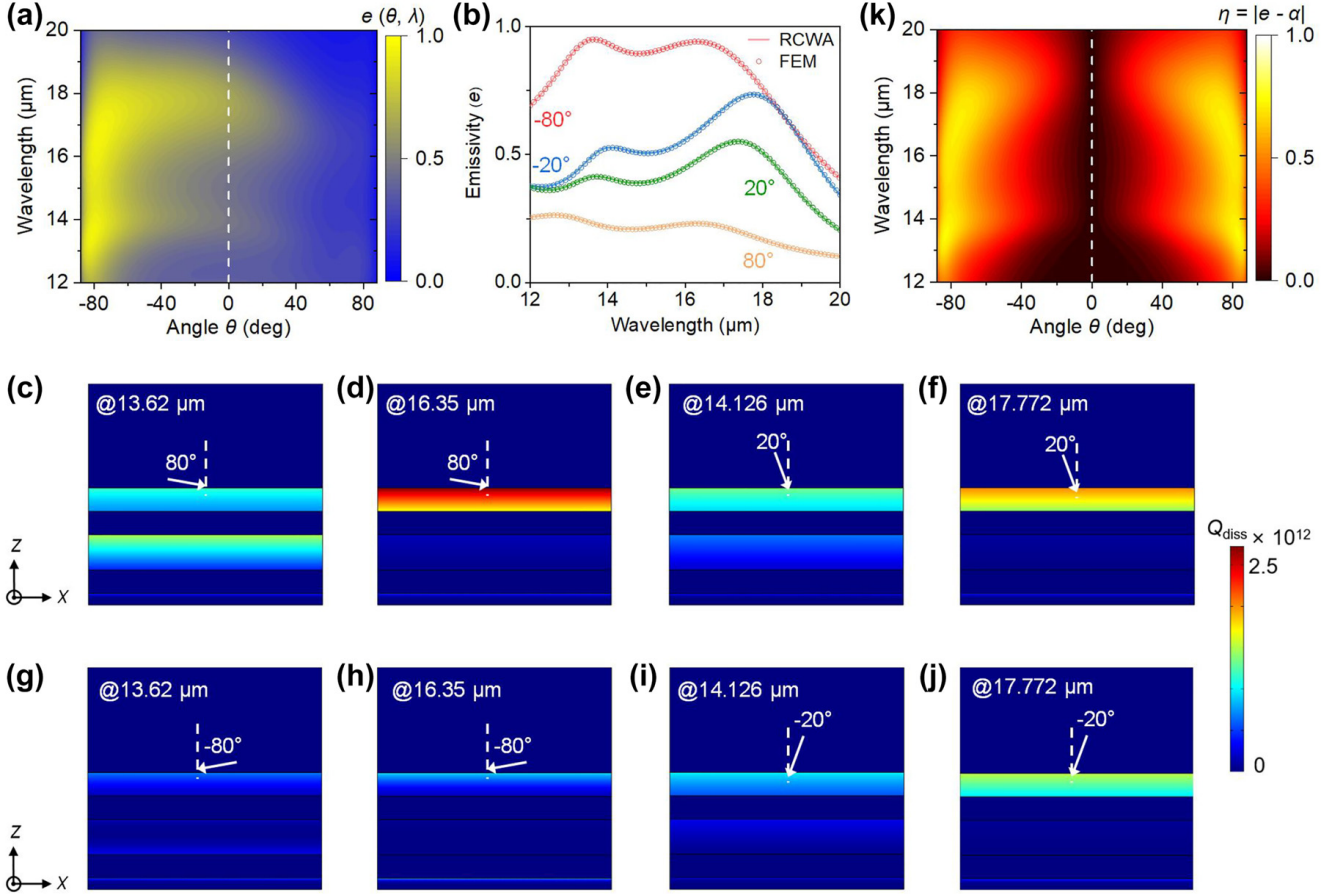
Performance and analysis of the nonreciprocal thermal emitter. (a) Contour map of *e* (*θ*, *λ*) of the WSM_1_/vacuum/WSM_2_/vacuum/Au multilayer structure across a wide wavelength range from 12 μm to 20 μm at different *θ* in plane *A* (*φ* = 0°). (b) Extracted emissivity values from (a) at some representative angles, including *θ* = −80°, −20°, 20°, and 80°. The hollow circles represent the FEM calculation results, while the color lines depict the results obtained through RCWA. (c)–(j) Distribution of electromagnetic power dissipation density within the multilayer structure at the corresponding wavelength, with *θ* set to ±80° and ±20°, respectively. (k) Contour map displaying the difference (*η*) between *e* (*θ*, *λ*) and *α* (*θ*, *λ*) of the multilayer structure. All the calculations are for the TM polarization, unless otherwise specified.

For simplicity, the dielectric films are initially assumed to be vacuum. We firstly focus on the calculation in plane *A* (with *φ* = 0°) unless indicated otherwise. Figure 3a illustrates the contour map of *e* (*θ*, *λ*) for our structure, ranging from 12 μm to 20 μm, with emission angles spanning from −88° to +88°. *e* (*θ*, *λ*) is extremely asymmetric in the whole angular range. Emissivity exceeding 0.5 is observed over a wide angular range and within an extremely broadband in the mid-infrared wavelength region. The noteworthy emissivity observed at large oblique angles, such as grazing angles, is primarily a result of the presence of two Brewster modes associated with ENZ materials at characteristic wavelengths. This emission profile can be broadened to encompass a wider spectral range due to the intrinsic loss characteristics of the two WSMs. Given that Brewster modes of the thin films are primarily excited at large angles, the inclusion of embedded dielectric layers in our design appears to effectively “retarding” the reduction in emissivity of these modes at smaller angles. Compare to the broadband nonreciprocal thermal radiation achieved at specific (large) angles when utilizing solely stacked WSMs [30] (without dielectric layers), we attribute the high emission of our structure at small angles primarily to the presence of dielectric layers, which effectively introduces an additional cavity. This facilitates extra reflections of electromagnetic waves within the cavity, thereby enhancing the absorption in the two adjacent WSMs. In view of this, the dielectric layer is supposed to be lossless in the wavelength from 12 μm to 20 μm, such as vacuum. Note that the enhanced emission at a small angle cannot be explained by Fabry–Perot (FP) resonance since the thickness of the dielectric layer we selected (*t*_2_ = 0.2 μm) does not satisfy the resonance conditions for FP resonance (where *t*_2_ = 4.403 μm would give resonance at *λ* = 14.126 μm and *θ* = 20°). Nevertheless, one can refer to some previous works in utilizing cavities to harness FP resonance for the thermal emission [49], [50].

The strong nonreciprocity exhibited by the two WSM layers results in a low emissivity in the opposite angular range with respect to normal direction. The spectral emissivity at *θ* = −80°, −20°, 20°, and 80°, is plotted in Figure 3b. The calculated emissivity values obtained through FEM are represented by the hollow circles, which show good agreement with the results (colored lines) obtained by RCWA. At *θ* = −80°, *e* (*θ*, *λ*) has an emissivity exceeding 0.9 over a broadband region (e.g., from 13.2 μm to 17.1 μm). However, as the emission angle approaches 0°, the influence of Brewster modes diminishes, resulting in a gradual decrease in the emissivity. The emissivity at each polar angle has two distinguishing peaks located at 13.62 μm and 16.35 μm when *θ* = −80°, similar to the Brewster wavelengths of pure WSM_1_ and WSM_2_. To provide a clearer physics insight underlying the peak emissivity, we have visualized the electromagnetic power dissipation density (referred to as *Q*_diss_) of the multilayer structure at a specific wavelength. Here, *Q*_diss_ (*x*, *y*, *z*) |_*θ*,__*λ*_ represents the spatial absorption/dissipation power density of the structure at a certain *θ* and *λ*, which is related to the absorptivity by the simple relationship:

(13) $$\alpha \left(\theta ,\lambda \right)=\frac{\int_{V\left(x,y,z\right)}Q_{\text{diss}}\left(x,y,z\right){|}_{\theta ,\lambda }dV}{P_{\text{incident}}{|}_{\theta ,\lambda }}$$

Based on equations (12) and (13), the distribution of *Q*_diss_ at specific *θ* provides an equivalent explanation for the emissivity observed at the opposite angle. We begin by considering *θ* = 80° as an example. At a specific wavelength of 13.62 μm, the power dissipation is influenced by two factors. The dominating factor is the power absorption by WSM_2_ at the bottom (Figure 3c), primarily due to its Brewster mode around 13.01 μm. However, WSM_1_ located on the top also contributes to the electromagnetic power absorption, owing to its intrinsic material loss that cannot be avoided. At a different wavelength of 16.35 μm shown in Figure 3d, the Brewster mode supported by WSM_1_ is capable of coupling with the free space electromagnetic field, resulting in the absorption of most of the power within the film near the ENZ wavelength. The gradual absorption, as indicated by the varying colors in WSM_1_, demonstrates the optimal thickness of the ENZ film for efficient energy capture.

For comparison, we have plotted *Q*_diss_ at the corresponding negative *θ* angles in Figure 3g and h at the respective wavelengths. The strong nonreciprocity, enhanced by the Brewster modes in the two WSMs, results in significantly weaker absorption at *θ* = −80°. This weaker absorption accounts for the much lower emissivity observed at *θ* = 80° in Figure 3b. A similar pattern is observed in the distribution of *Q*_diss_ at other incident angles, such as *θ* = 20° and −20° (Figure 3e, f, i, and j), despite a diminishing influence of the Brewster mode as *θ* approaches zero. Nonetheless, nonreciprocal emission at these small angles remains valid, facilitated by the presence of dielectric layers that effectively introduce an additional cavity, thereby enhancing emission/absorption at small angles.

Figure 3k presents the difference *η* between the emissivity *e* (*θ*, *λ*) and absorptivity *α* (*θ*, *λ*) of our structure, where larger values of *η* (i.e., closer to unity) indicate stronger nonreciprocity. Since *e* (*θ*, *λ*) = *α* (−*θ*, *λ*), it is expected that the contour map of *η* exhibits symmetry with respect to the normal direction. Across the angular range from −88° to 0°, a significant contrast between the emissivity and absorptivity is observed over a broadband region spanning from 12 μm to 20 μm, particularly within a large oblique angle range (e.g., from −88° to −60°) due to the contribution of the stronger Brewster modes. Despite the diminishing nonreciprocity as it approaches zero (i.e., normal incidence), a notable difference with *η* > 0.1 remains within the range of *θ* = −10° to −18°. This suggests that the robust nonreciprocal thermal radiation generated by the two WSM layers separated by the dielectric films can be extended to even smaller angles.

**Figure 4:**
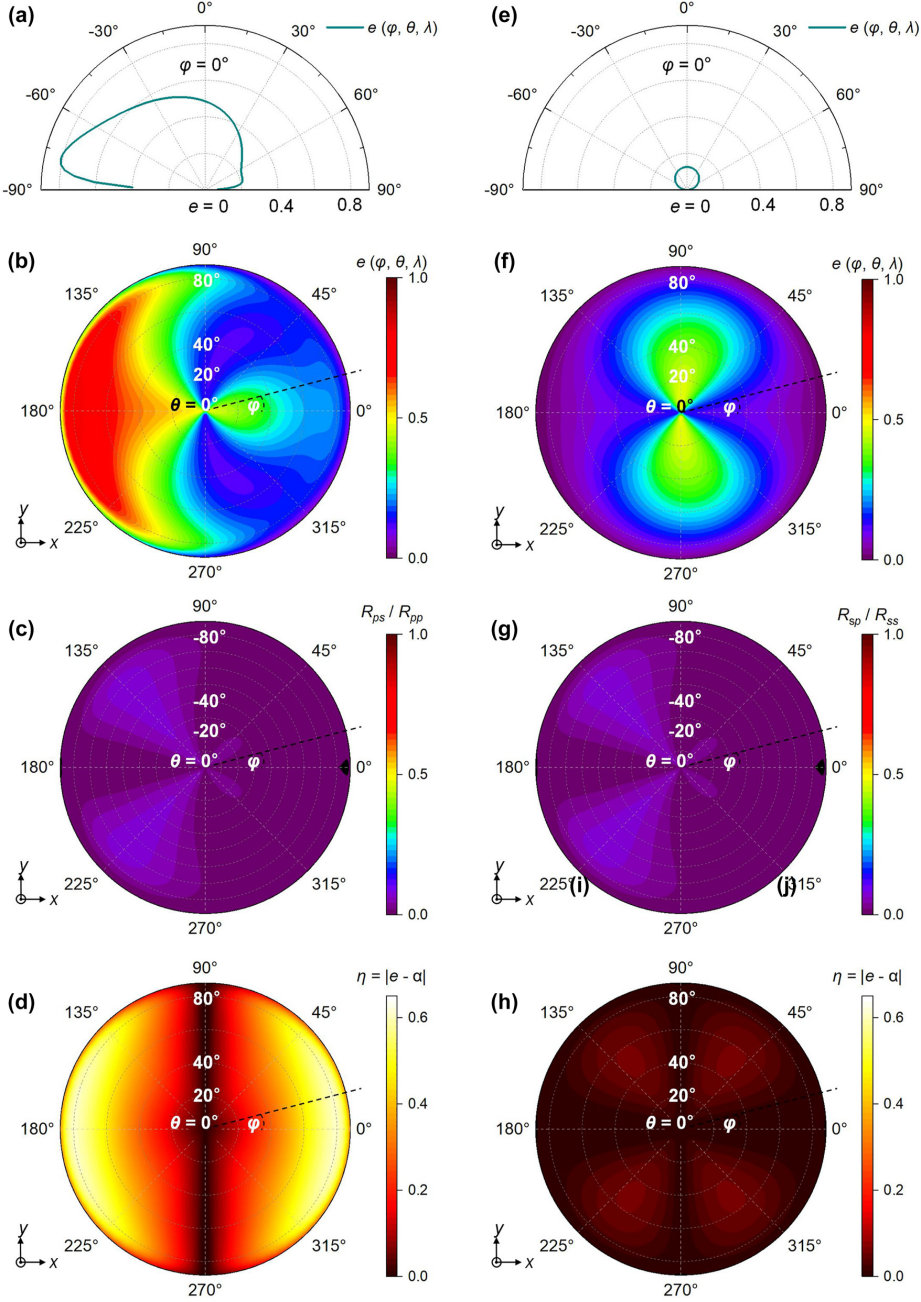
The calculations of emissivity, polarization conversions, and *η* for both TM polarization (a–d) and TE polarization (e–h), respectively. (a), (e): The average emissivity *e* (*θ*, *λ*) with *φ* = 0° across a broad wavelength range from 12 μm to 20 μm in polar coordinates, for the WSM1/vacuum/WSM2/vacuum/Au multilayer structure. (b), (f): Contour maps of the wavelength-averaged *e* (*φ*, *θ*, *λ*) of the multilayer structure in plane *A*′ with azimuth angle *φ* range from 0° to 360°. The radial direction represents the polar angle *θ* from 0° to 88°. The emissivity at *θ* = 0° in the plane with *φ* = 0° is given at a single spatial point. (c), (g): The ratio of Rps and Rsp to their respective polarizations (d), (h): The wavelength-averaged difference (*η*) between the emissivity and absorptivity in each plane, which is equivalently calculated by |*α* (*φ*, −*θ*, *λ*) – *α* (*φ*, *θ*, *λ*)|.

For the TM polarization, the average emissivity across a broad wavelength range (from 12 μm to 20 μm) is investigated (Figure 4a). The emission curve exhibits significant asymmetry at corresponding *θ* that are opposite in sign. The emissivity can exceed 0.8 at large oblique angles (e.g., −80°), while reaching 0.49 in the vertical direction. Conversely, emission in the other half-plane is relatively low and decreases with increasing *θ*, demonstrating the wide angular nonreciprocal thermal radiation of our structure. We further calculated the wavelength-averaged emissivity in different plane *A*′, where *θ* ranges from 0° to 88° (Figure 4b). The calculations incorporate polarization conversion in the reflectivity (denoted as *R*_*ps*_). With the equation *e* (*φ*, *θ*, *λ*) = 1 – *R*_*pp*_(*φ*, −*θ*, *λ*) – *R*_*ps*_(*φ*, −*θ*, *λ*), the minuscule ratio of *R*_*ps*_ to *R*_*pp*_ demonstrates its negligible contribution to the emission (Figure 4c). When *φ* is set to 0° or 180°, *e* (*φ*, *θ*, *λ*) corresponds to the results depicted in Figure 4a, with *θ* ranging from 0° to 88° or from 0° to −88°. Since the momentum separation 2**b** of the Weyl nodes aligns along the opposite of the *y*-direction, the nonreciprocity is strongest in the incident plane with *φ* = 0° at a certain *θ*. The nonreciprocity gradually diminishes to zero as *φ* approaches 90°, which is because there is no electric field component oscillating perpendicular to 2**b**, resulting in no nonreciprocal thermal radiation. Due to the nonreciprocity, the emissivity in the plane at *φ* (excluding 90° or 270°) differs from that in the plane at *φ* = *φ* + 180°. When *φ* is close to 0° or 180°, the difference becomes more pronounced over a wide angular range, particularly at large oblique polar angles, attributed to the stronger influence of Brewster modes. Although the plane at other non-zero *φ* is not completely perpendicular to the direction of 2**b**, the nonreciprocity remains robust over a considerable azimuth angle range, nearly a circle excluding *φ* = 90° or 270°.

The difference value *η* between the wavelength-averaged emissivity and absorptivity in each plane is calculated in Figure 4d, which is calculated by |*α* (*φ*, −*θ*, *λ*) – *α* (*φ*, *θ*, *λ*)| based on equation (12). Following the relationship: *η* (*φ*, *θ*, *λ*) = *η* (*φ* + 180, *θ*, *λ*), the absolute value of *η* exhibits symmetry with respect to the normal direction (*θ* = 0°) in each plane with a non-zero *φ*. The wavelength-averaged *η* can reach up to 0.6 at large oblique polar angles across a wide range of azimuth angles. It remains distinguishable from *θ* = 16°–88°, indicating the robustness of the nonreciprocal thermal radiation of the emitter.

We have also conducted calculations to assess the performance of TE polarization, as depicted in Figure 4(e)–(h). As anticipated, no nonreciprocity is observed in the TE mode within the plane where *φ* = 0°. The contour map of *e* (*φ*, *θ*, *λ*) in Figure 4(f) further illustrates the absence of noticeable nonreciprocity when *φ* is not equal to zero. This can be attributed to the weak polarization conversion *R*_*sp*_, as indicated in Figure 4g. Similarly, considering that *e* (*φ*, *θ*, *λ*) = 1 – *R*_*ss*_(*φ*, −*θ*, *λ*) – *R*_*sp*_(*φ*, −*θ*, *λ*), the minimal ratio of *R*_*sp*_ to *R*_*ss*_ underscores its negligible contribution to the emission. Given that both *R*_*ss*_ and *R*_*sp*_ exert little influence on nonreciprocity, the different value *η* of TE polarization between the wavelength-averaged emissivity and absorptivity remains minimal. Despite the relatively lower nonreciprocal contribution of the TE modes, the broadband and wide-angle nonreciprocal thermal radiation persists even after averaging the TE and TM modes. One may also employ a TM polarizer to eliminate the influence of the TE modes [26], [27].

**Figure 5:**
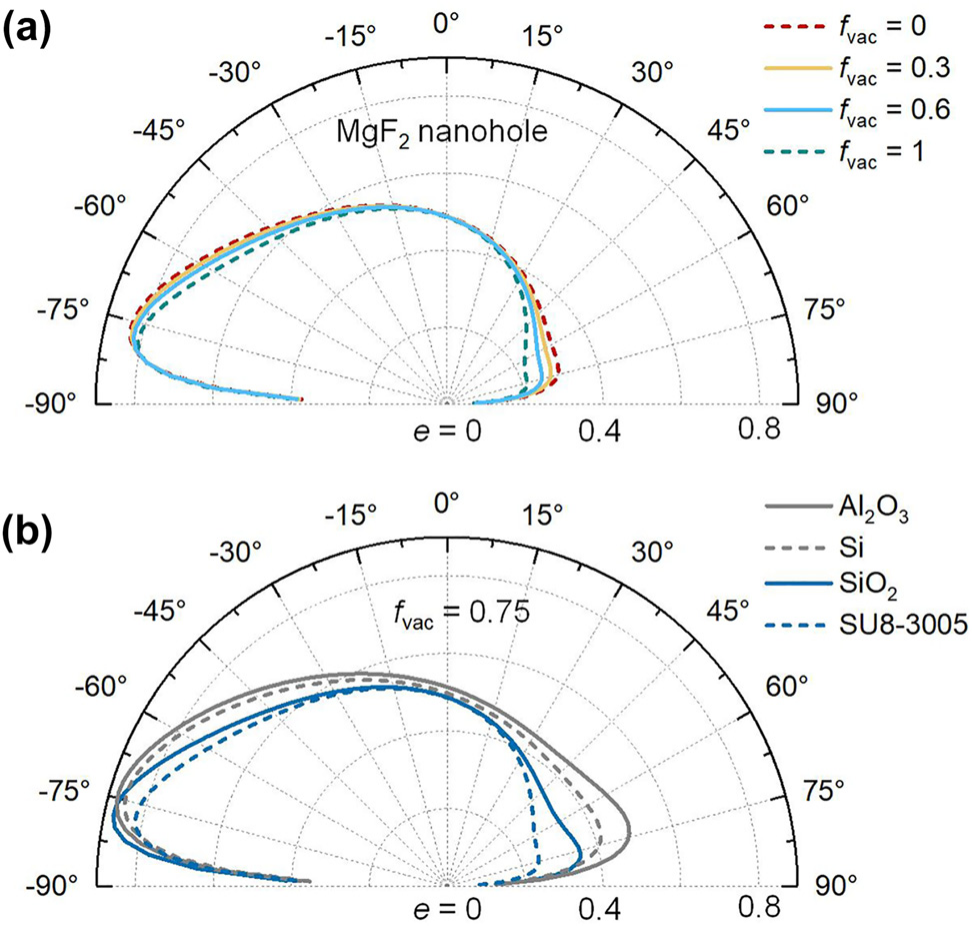
Robustness analysis of the nonreciprocal thermal emitter. (a) Average Emissivity *e* (*θ*, *λ*) of a multilayer structure with the dielectric layers replaced by some MgF_2_ nanohole structures with different vacuum filling ratios *f*_vac_. The calculated wavelength ranges from 12 μm to 20 μm. (b) Corresponding average *e* (*θ*, *λ*) of the multilayer structure with the dielectric layers are Al_2_O_3_, Si, SiO_2_, and SU8-3005 layers, respectively. The calculations are for TM polarization.

From a practical perspective, the utilization of specific materials or nanostructures as dielectric layers are discussed. In the context of these structural parameters, one can anticipate the presence of materials exhibiting a near-unity refractive index and minimal loss, such as MgF_2_ [51] or SU8-3005 [17], or a planar layer of nanohole structure with an appropriate vacuum filling ratio. Figure 5a presents the wavelength-averaged emissivity of the multilayer structure when the dielectric layers are replaced with a nanohole structure made of MgF_2_. Since MgF_2_ is optically similar to vacuum within the considered wavelength range, even with a vacuum filling ratio of 0, the emissivity remains similar to that of the WSM_1_/vacuum/WSM_2_/vacuum/Au multilayer structure shown in Figure 4a. Increasing the vacuum filling ratio brings the emissivity closer to that of the vacuum case, as demonstrated by the blue-solid line in Figure 5a for a vacuum filling ratio of 0.6.

Other materials such as Al_2_O_3_, Si, SiO_2_, and SU8-3005 are examined as nanohole structures with a vacuum filling ratio of 0.75 in Figure 5b. The permittivity data for these materials are obtained from previous experimental results [52], [53]. Irrespective of the optical properties of the dielectrics, the nanohole structures with a suitable vacuum filling ratio allow the multilayer structure to showcase the desired nonreciprocal thermal radiation performance. The remarkable nonreciprocity observed for the TM polarization is maintained across a wide range of angles and over a broadband. Among the tested materials, the Al_2_O_3_ nanohole structure exhibits the highest emissivity across the angle range from −88° to 0°, but it is accompanied by moderate (not small) emissivity in the opposite angle range, which may not be favorable for achieving nonreciprocity. Therefore, a balance between high emissivity and large nonreciprocity needs to be considered. The emissivity in the case of the SU8-3005 closely resembles the vacuum case, even with a filling ratio of 0. Therefore, if the dielectric layers are optically similar to vacuum or some nanohole structures with an appropriate vacuum filling ratio, the multilayer structure can exhibit robust broadband and wide angular nonreciprocal thermal radiation. Note that the selection of permittivity close to that of a vacuum may not be the only option, particularly when structural parameters are subject to change. Other lossless materials, such as germanium (Ge) with a higher permittivity, may also be considered but requiring additional optimization of structural parameters (not shown). Moreover, certain pyrochlore iridates, such as Eu_2_IrO_7_ [39], [54], can exhibit the parameters used in this calculation. The gradient chemical potential can be experimentally adjusted through doping, where the types and concentrations of dopants are controlled [30], [55]–[57]. The fabrication of WSM can be achieved through a solid-state reaction technique [58]. Furthermore, recent experiments have started to showcase the feasibility of growing WSM thin films. For instance, the growth of tantalum-arsenide (TaAs) thin films was demonstrated using molecular beam epitaxy and pulsed laser deposition techniques [59], [60]. These methods may also potentially be applicable to other WSMs, such as Eu_2_IrO_7_. The dielectric and metal films can be fabricated using standard atomic layer deposition or E-beam evaporation methods [17].

## 3 Conclusions

In conclusion, we propose a mid-infrared ultra-broadband wide angular nonreciprocal thermal emitter by constructing a robust nonreciprocal system using Weyl semimetal metamaterials. For the TM polarization, by harnessing the ENZ-induced Brewster modes and the strong nonreciprocity arising from the two WSMs with gradient Fermi levels, our emitter exhibits significantly nonreciprocal radiation over a broad wavelength range from 12 μm to 20 μm, spanning a wide angular range from 16° to 88°. The nonreciprocal thermal radiation is achieved without the need for an external magnetic field and provide an ultra-wide angular range within the mid-infrared band. The wavelength-averaged emissivity, considering the full azimuth angle *φ* range from 0° to 360°, confirms the robustness of the emitter’s broadband and wide angular nonreciprocal emission. The performance of the TE polarization has also been investigated but exhibits negligible nonreciprocity. Furthermore, we explore the utilization of alternative practical materials and nanostructures as dielectric layers inserted between the two WSMs, showcasing the flexibility of our design scheme and the reliable performance of the nonreciprocal emitter. This research not only provides valuable insights for the experimental design of nonreciprocal thermal emitters but also holds promise for various applications in fields such as higher-efficiency radiative cooling, medical sensing and infrared heating, energy conversion, thermal management, and directional thermal camouflage.
